# 39 Silent Scars: Distinguishing the Psychiatric Morbidities Following Burn Injuries Between Males and Females

**DOI:** 10.1093/jbcr/iraf019.039

**Published:** 2025-04-01

**Authors:** Matthew Dao, Joshua Lewis, Mbinui Ghogomu, Blancheneige Beohon, Philong Nguyen, Steven Wolf, Amina El Ayadi, Juquan Song

**Affiliations:** University of Texas Medical Branch; University of Texas Medical Branch; University of Texas Medical Branch; University of Texas Medical Branch; University of Texas Medical Branch; University of Texas Medical Branch; University of Texas Medical Branch; University of Texas Medical Branch

## Abstract

**Introduction:**

Burns have a profound impact on long-term psychiatric health. However, gender differences in mental health outcomes following burn injuries remain unclear. This retrospective cohort study aims to investigate gender differences in mental health co-morbidities among burned patients.

**Methods:**

Adult burn patients (≥18 years) were identified using the TriNetX Network database and were stratified by gender. Mental health outcomes, including post-traumatic stress disorder (PTSD), anxiety, depression, suicide ideation and attempts, adjustment disorders, and substance use disorders were assessed at both 3 months and 1-year after burn. Risk ratios (RRs) were used and significance was set at p< 0.05 with 95% confidence intervals.

**Results:**

TriNetX identified 248,919 women and 261,985 men with burns across 63 healthcare organizations. We performed the propensity score matching between two groups for age, race, ethnicity, and burn severity. At 3 months, women exhibited significantly higher risks of anxiety (RR = 1.545) and depression (RR = 1.394) but presented significantly lower risks of suicide attempts (RR = 0.789) and substance use disorders (RR = 0.568) compared to men. At three months, PTSD and adjustment disorders were not significantly different. At the 1-year mark, women had significantly higher risks of anxiety (RR = 1.805), depression (RR = 1.587), PTSD (RR = 1.263), and adjustment disorders (RR = 1.351) but lower risks for suicide attempts (RR = 0.901) and substance abuse disorders (RR = 0.697) compared to men (p< 0.05).

**Conclusions:**

Significant gender differences in mental health outcomes were identified from 3 months to 1 year following burn injuries, with women more prone to anxiety, depression, adjustment disorders, and PTSD, and men more susceptible to suicide attempts and substance use disorders. These findings emphasize consideration for gender-specific mental health interventions in burn care.

**Applicability of Research to Practice:**

Incorporating gender-specific psychiatric care in burn rehabilitation should be considered.

**Funding for the Study:**

This research was funded by a Clinical and Translational Science Award (UL1 TR001439) from the National Center for Advancing Translational Sciences at the NIH.

